# Supplementary material for: Transcriptome Analysis Reveals Differentially Expressed Genes Involved in Cadmium and Arsenic Accumulation in Tea Plant (Camellia sinensis)
Source: Plants (Basel). 2023 Mar 5;12(5):1182. doi: 10.3390/plants12051182 (PMC10007383; doi:10.3390/plants12051182)
Supplement: Supplementary file 1 [file plants-12-01182-s001.zip › Tables.zip!/Table S1.docx]

**Supplementary Table S1 | Primers used in this study.**

| **Gene** | **Forward primer** | **Reverse primer** |
| --- | --- | --- |
| CSS0050491 | CAGTCCATATCCCATGATGA | CGACTCACATAGTCCCTAAA |
| CSS0019367 | GAACCTAGTCTCCCAATTTATG | TTAGAGAAAGAAAGGGTGGT |
| CSS0033791 | GTCTACTTCAACAACCGATTAC | ATCATACCTACCCACAGTTC |
| CSS0001107 | CCTACTATGCCATCAACTACTC | GGATTGTCAAAGAGGTGATCTA |
| CSS0035212 | GAAGAGTTGGTGGGACATATAC | TATGACTTTGTCGGCTTCAC |
| CSS0006162 | GAAATCAGTGGATGTGGATTTG | GGTGAGCTATCAGGTTGTATG |
| CSS0004428 | GGGAGACAGTGTTTAGTGTAAG | CAGTTGAAACTCTTCCTTGTTG |
| CSS0000647 | CCAAGAGGAAGGATGATGATG | GTAGAGCTTGGTAGGTGAAATAG |
| CsGAPDH | ATGATAACTGAAAAAACAAATGTAA | TTAAGCTTCATAAACCTTGCACT |
