# Supplementary material for: Transcriptome Analysis Reveals Differentially Expressed Genes Involved in Cadmium and Arsenic Accumulation in Tea Plant (Camellia sinensis)
Source: Plants (Basel). 2023 Mar 5;12(5):1182. doi: 10.3390/plants12051182 (PMC10007383; doi:10.3390/plants12051182)
Supplement: Supplementary file 1 [file plants-12-01182-s001.zip › Tables.zip!/Table S10.docx]

**Supplementary Table 10 |** Coding DNA Sequence of eight candidate genes in *C*. sinensis.

>CSS0000647	PREDICTED:Camellia sinensis ethylene-responsive transcription factor ERF054-like

ATGGATCAATCTGGGAATAGTGGCAAATCAAAAAAAGTTATTGAAGAAACTGAGAAGATGGCCCAGATGGAATGGGTGAGAGACCAAACTAAGGACTTCGACATCGGCTTCGAGAGGCCACAATGGAAGCCAGTTTTTGAAGAAGCTTCCATGTCAAGTAGGCCTCTCAAGAAGGTCAAAAGTCCTGAACGCCAAGACCCATTTCAATCCTCTGTTTCTTCAGCTCAACAAACTCCTTCTCTGTCTCTCTCATCCTCTGTTCCTTACACTACTACAGCACCCATAGCTTTCCCTCCATCATCATCTTCAAGGCTTGTATTTCCTTTTGCCTTGGATAATTCTCAACAATCCATGGAAAATCTACACCAATTCCAAACCAACCCGCCTCCTCTGTTCCTTCAACCGCCACAAAATCAACAGCAAATGATTTCCTTTGCTCCTCAGAACCAAAGCCTAGGCTTCCCACCATACTTTGCCGGAGACTCGGCTCCATCACAGCAGCAATTACTGCAGTACTGGAGTGATGCACTGAATTTGAGTCCAAGAGGAAGGATGATGATGATGAACAGGTTGGGACAGGATGGTCGGGGCTTGTTTAGGCCTCCAGTGCCCCCTATTTCACCTACCAAGCTCTACAGAGGAGTGAGGCAGAGACATTGGGGCAAATGGGTAGCTGAAATTCGTCTCCCTCGAAACAGGACTCGCCTCTGGCTCGGCACCTTTGACACAGCAGAGGACGCCGCCTTAGCCTATGATCGCGAGGCCTTTAAGTTGAGGGGAGAGAATGCACGGCTCAATTTCCCCGAACTCTTCCTCAATAAAGACAGAGCAGCTTCGTCAGCGCCCGGTTCATCTTCATCGTCTCCTCCTACTCCCCATGGAAATTTGGCTCCTAGCCGGTACTCGAAACAACCTCAGGAAGCTTCAGAAGGCCCAAATTTGGAGGTTTCAAGTATGGAAATGCCACCACCAGAGTGGCCCAAACCTCAAGAAGATAACCCAGATGAGGATTCGGGATTGGGGTCGAGCGAGGTCACAGTTGCAGGGGGTTCTGGAGCAGGGGAAGGTATTTTGGAGTCATCGGAATTGGTTTGGGGTGACATGGCAGAGGCTTGGATGAATGCAATTCCAGCTGGTTGGGGTCCAGGTAGTCCAGTTTGGGATGATTTGGACACAACCAATAATCTTCTGTTGCCTTCAAATCTTCCTTTTGCCAATGTTCATCAGCAGGACTTGAGTGGTTCTAATCCTCAAAAACAGGAACAGAACATCGCCCCGGCTGATTCATCGTCTTCTTCTTCTTCATGCCCTATGAAGCCCTTCTTTTGGAAGGATCAGGACTGA

>CSS0033791	PREDICTED:Camellia sinensis 9-cis-epoxycarotenoid dioxygenase NCED1

ATGGCTTCTTCAACAACACCTTCTACAAGTACATGGGTTAAGCCCAAATTGATCCCTACTTCCTCGAGTGCATTGGGCTCTTGTTCCACCTCCTCATTCTCTCTAAGCAAGCCCAATCCCAATAGGCAACAAAACATTGCTTGCTCTCTCCACACCCCTTCCATACGCCACCTTCCAAAGCACGCTCCCCCAACATATCACACACTCAGCACTGGTATTGCCACTAAAGATAACTCTTCCTTGTCCTCCACCTCTGCCAATGCTCAATCATGGAATTTCTTACAGAAAGCAGCGGCAATGGCGTTGGATGTAGCGGAGAGTATCCTAGTGGCACGCGAAAAAGAGCATCGTCTACCCAAGACGGCCGACCCGGGAGTCCAAATTTCCGGCAACTTCGCCCCGGTGCCGGAGCACTCGGTCCAGCACAACCTCCGCATCGCCGGAAAAATCCCCGAATGCATTCAAGGCGTTTACATCCGGAACGGAGCGAACCCGCTATTCGAGCCGGTTGCCGGCCACCACTTCTTCGACGGCGACGGCATGGTCCACGCCGTCAAATTCAATGACGGCGCGGCTAGCTACTCCTGCCGGTTCACCGAAACCCAGAGGCTCGTTCAGGAACGGGCCTTGGGCCGGCCCGTCTTCCCCAAAGCCATCGGCGAACTCCACGGCCACTCCGGCATCGCCAGGCTATTACTGTTCTACGCTCGCGGGATATTCGGGCTCGTCGATCACAGCCACGGCACTGGCGTAGCCAACGCCGGACTAGTCTACTTCAACAACCGATTACTCGCCATGTCGGAAGACGATTTGCCGTACCACGTCAGAGTCACCCCCTCCGGCGATCTCAGAACTGTGGGTAGGTATGATTTCGATAATCAACTCAATTCCACCATGATCGCTCATCCCAAACTCGACCCAGTTTCCGGCCAACTCTTCGCTCTCAGCTACGATGTGATCCAGAAACCATACCTCAAATACTTCAAATTTTCACCCTCCGGCGAGAAATCACCGGACGTTGAAATCCCACTCTCAGAACCAACCATGTTGCATGATTTCGCAATCACCGAGAAATTCGTAGTGATTCCTGATCAACAAGTAGTTTTCAAGCTCCCGGAGATGATCCGCGGTGGCTCGCCGGTGGTTTACGACAAAAACAAGATGTCGAGGTTTGGGATTTTGAACAAGAATGCCACTGATGCTTCTGAGATCAAATGGGTCGAAGCACCAGATTGCTTTTGCTTCCACCTCTGGAATGCTTGGGAAGAGCCAGAGACCGATGAGATCGTCGTGATTGGGTCTTGTATGACTCCACCGGACTCAATTTTCAACGAATGTGACGAGGGACTAAAGAGTGTTCTATCAGAAATCAGGTTCAATTTAAAGACTGGTAAGTCTTCTCGCAGACCCATAATGAGTGAGACTGATCAAGTGAATTTAGAGGCAGGAATGGTGAACCGGAACTATCTCGGCCGCAAATCCCAGTTCGCTTACCTCGCCATCGCCGAGCCGTGGCCTAAAGTCTCCGGATTCGCCAAAGTCGACCTCTCCACTGGCGAGGTGAAGAAACACAATTACGGCAATGAAAAATACGGTGGCGAGCCTCTGTTCCTGCCCAGAGACCCAAATTCAGTATCTGAAGATGATGGGTATATTCTAGCTTTTGTACACGACGAGAAGAAGTGGAAATCAGAGCTACAGATCGTGAACGCCATGAATTTAGAATTAGAAGCTACAATCGAGCTACCATCTCGAGTTCCCTATGGCTTTCATGGCACATTCATAAGTGCCAAAGACTTAGCCAAACAAGCCTAA

>CSS0050491	PREDICTED:Camellia sinensis probable galactinol--sucrose galactosyltransferase 5

ATGGCTCCAAGTTTGAGCAAAGCTGGCTCCGATATTTCAGACCTCGTCAATGGCTACACCGGCTCATCATCACCAACAATCACATTAGAGGGATCAAACTTCAAAGCCAGAGGCCACGTCATTCTCTCCGATGTACCTCCCAACATCGTAGCTTTTCCCTCACCCTACGGCGGCGGCGCCACCACCACCGCCGCCGGCAGCTTCGTCGGATTCGACTCCCGCGAGGCCAAAAGCCGCCACGTGGTGTCGGTGGGCAAGCTCAAGGGGATCACATTCATGAGTATTTTCAGATTCAAGGTCTGGTGGACGACCCACTGGGTCGGATCCAACGGCCGAGACCTCGAATCCGAAACCCAGATGGTGATTCTGGACCGCTCAGCCGAGGGCCGCCCTTACGTCCTTCTCCTCCCCCTCCTTGAAGGCCCATTCCGCGCCTCTCTCCAACCCTGCGGGTCGACAAAAGTGACCCGAGCTTCGTTTCGGAGCGTGCTTTACATTCACGCAAGCGACGATCCGTACAGTCTCGTGGAGGACGCCATGGAAGTAGTGAGAGCTCATCTCGGAACATTCAGGCTTTTGAAAGAGAAGACACCACCGGGTATCGTGGACAAATTTGGTTGGTGCACGTGGGATGCATTTTACCTTACGGTGCAACCTCAGGGAGTTTGGGAAGGTGTGAAAGGGCTCGTAGAAGGCGGGTGCCCACCGGGTCTGGTTCTTATTGATGATGGGTGGCAGTCCATATCCCATGATGATGACCCGATTGCCCAAGAAGGCATGAATCGGACCTCTGCTGGTGAGCAAATGCCGTGTAGGCTCATAAAGTTTGAAGAGAATTACAAATTTAGGGACTATGTGAGTCGTAAAAAGTCAACCTCTGGTGGTGCCCTTAATAAGGGCATGGGTGCCTTTATTAGGGACCTCAAGGGGGAGTTTAAGAGTGTGGATTATGTGTATGTGTGGCATGCTTTGTGTGGGTATTGGGGCGGGCTTAGACCCAATGCTTCGGGTCTACCCAAATCTAGAGTTGTGAGGCCTAAGTTGTCACCTGGGTTGGAGAAGACAATGGAAGATCTAGCCGTTGATAAGATCGTGAACAATGGAGTTGGGTTGGTCCCACCAGAGATGGTTGATCAAATGTATGAAGGTCTGCACTCGCATTTGGAGTCAGTTGGGATTGATGGAGTCAAAGTGGATGTTATTCACTTGTTGGAGATGTTGTGTGAGGACTATGGAGGCAGAGTAGAACTAGCAAAAGCATACTACAAAGCATTGACATCTTCAGTGAGGAAGCACTTCAATGGCCATGGTGTCATCGCAAGCATGGAACACTGCAACGACTTCATGTTCCTCGGAACCGAAGCCATAGCTCTCGGTCGTGTCGGGGATGATTTTTGGTGCACTGATCCATCTGGGGATCCCAATGGGACCTTTTGGCTACAAGGGTGTCACATGGTGCACTGTGCTTATAACAGCTTGTGGATGGGGAACTTCATACACCCAGATTGGGACATGTTCCAATCCACTCATCCTTGTGCCCAGTTCCACGCCGCCTCTCGGGCTATTTCCGGTGGACCAATTTATGTCAGCGACTCTGTCGGAAAACACGATTTCAAACTGCTCAGGAGCCTGGTGTTACCTGATGGCTCGATCTTGCGGTGCGAGCACTATGCACTTCCTACCCGTGATTGTCTCTTCCAAGACCCTCTCCACGATGGGAAGACCATGCTCAAGATATGGAACTTAAACAAGTACACTGGAGTTCTTGGAGCATTTAACTGCCAAGGTGGAGGATGGTGTCGCGAGTCCAGGCGCAACAAATGCGCCTCCGACTGTTCCCACACCGTGACCACAACCACCACCCCAAAAGACATTGAGTGGAAACGTGGAACAAAGCCAATCTCCATTGAAGCAGTGCAACTATTTGCATTGTACATGTTCCGCGAGAAAAAATTAATTCTCTCAAAGCCATCCGACGCCATAGAGATCTCACTCGAACCCTTTAACTTTGAGCTTATAACGGTCTCTCCAATCACCACCATGGCTAACAAGTCTATCCAATTTGCTCCGATTGGGCTGGTGAATATGCTTAACACTGGCGGTGCAATACAGTCAGTGGTGTTCAATGATCGGGACAACTCGGTCCAGGTAGGGGTGAAGGGGACTGGTGAAATGAGGGTGTTTGCGTCGGAGAAACCGACAGCTTGCCGGCTTAATGGGGAAGATGTTGAATGTGGTTATGAGGGGCTCATGGTTGTAGTTCAAGTGCCATGGCCTGAATCTTCAAGTTTGTCTATTATTGAGTACTCATTTTAA

>CSS0001107	PREDICTED:Camellia sinensis galactinol synthase 2-like

ATGGCTCCTGAACTTGTGAGTGCAACCACCAAGGCCACCAGCCTAGTCAAGGCCGCGAGCCTGATGAGCAGAGCTTATGTCACTTTCTTGGCTAGCAATGGTGACTATGTCCAGGGCGTGGTCGGCCTAGCCATGGGCCTGAGGAAGGTCCACACTGCCTACCCGCTTGTGGTGGTCGTGCTTCCGGATGTGCCGGAGGAACACCGCCGTATGCTGGTGGCTCAGGGATGCATAACCCAGTTTGCTATGGCCTACTATGCCATCAACTACTCCAAGCTTCGCATCTGGGAGTTTGTGGAATACCATAAGATGATATACTTGGACGGAGACATCCAGGTGTTTGAAAACATAGATCACCTCTTTGACAATCCAAATGGCTACTTTTATGCTGTGAAGGACTGTTTTTGTGAAAAGACATGGTCTCACACCCCACAGTACCAGATTGGGTACTGCCAACAGTGTCCTGATAAGGTCCAGTGGCCTGCAGAGTTGGGTCCCAAGCCTCCCCTCTACTTCAATGCTGGCATGTTCATGTTCGAGCCCAGTCTCTCAATTTATGATGACCTCTTAGAGACCCTCAAAATCACCCCGCCTACCCCTTTTGCTGAGCAGAAAAACCCACAAACTCACCAAAGCATCCCCACATTTCTTCTTCATTTTCTCAAAAACACTTCCCAACTTCCTTTCTCTCTCTACAGACCTCTCTTTCTTTCTCTAAAACTTGTTGTGAAAACTCAAATGGCTCCTGAACTTGTGAGTGCAACCACCAAGGCTACCAGCCTGGTCAAGGCAGCCAGCCTGACGAGCCGGGCTTATGTGACATTCTTGGCAGGGAATGGTGACTACGTGAAGGGCGTGGTTGGGCTGGCCAAGGGCCTGAGGAAGGTCAAGACTGCGTACCCGCTAGTGGTGGCGGTCCTGCCTGACGTGCCTGAGGAGCACCGCCGTATGCTGTTAGCTCAGGGCTGCATAGTCCGTGAGATCGAGCTGGTGTACCCGCCAGCGAACCAGACCCAGTTTGCTATGGCCTACTATGTCATCAACTACTCCAAGCTTCGTATCTGGGAGTTTGTGGAGTACCATAAGATGATATATTTGGACGGAGACATCCAGGTGTTTGAAAATATAGACCACCTCTTTGACAACCCAAATGGCTACTTCTATGCTGTGAAGGATTGTTTCTGCGAGAAGACATGGTCTCATACCCCACAGTACCAGATTGGGTACTGCCAACAGTGCCCTGATAACGTCCAGTGGCCTAGAGAGTTGGGTCCTAAGCCTCCCCTCTACTTCAATGCTGGCATGTTTGTGTTCGAGCCTAGTCTCCCAATTTATGATGACCTCTTAGAGACCCTCAAAATCACTCCGCCTACCCCTTTTGCTGAGCAGGACTTTTTGAATATGTTCTTTAAGGATGTCTACAAGCCTATCCCACCAATTTACAACCTTGTTTTGGCCATGCTTTGGCGTCACCCAGAAAACATTGAACTTAATAAAGTGAAAGTTGTTCACTACTGTGCCGCGGGATCAAAGCCATGGAGGTATACTGGAATTGAAGAGAACATGAAAAGGAAAGACATTAAGATGCTAGTGAAGAGCTGGTGGGATATATACAGTGATGAGTCATTAGATTATAAGAAGACTAACACATCGCCCATTGAAGGTGAAGCTGACAAATTCATGGCGGCAACGGTGTCAGAGGCTAACGCTGGTCATTACATTACCGCCCCATCTGCTGCCTAG

>CSS0019367	PREDICTED:Camellia sinensis galactinol synthase 2-like

ATGGCTCCTGAACTTGTGAGTGCAACCACCAAGGCTACCAGCCTGGTCAAGGCAGCCAGCCTGACGAGCCGGGCTTATGTGACATTCTTGGCAGGGAATGGTGACTACGTGAAGGGCGTGGTTGGGCTGGCCAAGGGCCTAAGGAAAGTCAAGACTGCGTACCCGCTAGTGGTGGCGGTCCTGCCTGACGTGCCGGAGGACCACCGCCGTATGCTGCTAGCTCAGGGCTGCATAGTCCGTGAGATCGAGCCGGTGTACCCACCGGCGAACCAGACCCAGTTTGCTATGGCCTACTATGTCATCAACTACTCCAAGCTTCGTATCTGGGAGTTTGTGGAGTACCATAAGATGATATATTTGGACGGAGACATCCAGGTGTTTGAAAATATAGACCACCTCTTTGACAACCCAAATGGCTACTTCTATGCTGTGAAGGATTGTTTCTGCGAGAAGACATGGTCTCATACCCCACAGTACCAGATTGGGTACTGCCAACAGTGCCCTGATAAGGTCCAGTGGCCTAGAGAGTTGGGTCCTAAGCCTCCCCTCTACTTCAATGCTGGCATGTTTGTGTTCGAACCTAGTCTCCCAATTTATGATGACCTCTTAGAGACCCTCAAAATCACTCCGCCTACCCCTTTTGCTGAGCAGACCACCCTTTCTTTCTCTAAAACTCAAATGGCTCCTGAACTTGTGAGTGCAACCACCAAGGCTACCAGCCTGGTCAAGGCAGCCAGCCTGACGAGCCGGGCTTATGTGACATTCTTGGCAGGGAATGGTGACTACGTGAAGGGCGTGGTTGGGCTGGCCAAGGGCCTGAGGAAGGTCAAGACTGCGTACCCGCTAGTGGTGGCGGTCCTGCCTGACGTGCCGGAGGACCACCGCCGTATGCTGCTAGCTCAGGGCTGCATAGTCCGTGAGATCGAGCCGGTGTACCCACCGGCGAACCAGACCCAGTTTGCTATGGCCTACTATGTCATCAACTACTCCAAGCTTCGTATCTGGGAGTTTGTGGAGTACCACAAGATGATATATTTGGACGGAGACATCCAGGTGTTTGAAAATATAGACCACCTCTTTGACAACCCAAATGGCTACTTCTATGCTGTGAAGGATTGTTTCTGCGAGAAGACATGGTCTCATACCCCACAGTACCAGATTGGGTACTGCCAACAGTGCCCTGATAAGGTCCAGTGGCCTAGAGAGTTGGGTCCTAAGCCTCCCCTCTACTTCAATGCTGGCATGTTTGTGTTCGAGCCTAGTCTCCCAATTTATGATGACCTCTTAGAGACCCTCAAAATCACTCCGCCTACCCCTTTTGCTGAGCAGGACTTTTTGAATATGTTCTTTAAGGATGTCTACAAGCCTATCCCACCAACTTACAACCTTGTTTTGGCCATGCTTTGGCGTCACCCAGAAAACATTGAACTTAATAAAGTGAAAGTTGTTCACTACTGTGCCGCGGGATCAAAGCCATGGAGGTATACTGGAATTGAAGAGAACATGAAAAGGAAAGACATTAAGATGTTAGTGAAGAGCTGGTGGGACATATACAATGATGAGTCATTGGATTATAAGAAGACTAACACATCACCCATTGAAGGTGAAGCTGACAAATTCATGGCGGCAACGGTGTCAGAGGCTAACCCTGGTCATTACATTACCGCCCCATCTGCTGCCTAG

>CSS0006162	PREDICTED:Camellia sinensis heavy metal-associated isoprenylated plant protein 23-like

ATGGGAGTTTCAGGCACTTTGGAGTACTTCTCTGAATTGCTGAGCAGTGTCAAGAAGGTCAAGAAAATGAAACAGTTAAACACTGTGGCTCTCAAAGTCAGGATGGACTGTGAGGGTTGTAAACGTAAAGTCAAAAACACACTCTCTTCTCTCAAAGGAGTGAAATCAGTGGATGTGGATTTGAAGGAGCAGAAGGTGACAGTGAGTGGATATGTGGATGCAAAGAAAGTGCTGAAGAAGGCTCAATCTACTGGTAACAAATCTGAGCCATGGCCATATGTTCCATACAACCTGATAGCTCACCCATACGCTGCTCAAGTTTATGACAAGAAGGCACCTCGTGGTTTTGTTAGGGGCACTGAAGACACTGCCATTGCTACTCTCAGCACGGTTGAACAACACTACACCACCATGTTCAGTGATGATAATCCTAATGCTTGCTCTATCATGTAGC

>CSS0004428	PREDICTED:Camellia sinensis calcineurin B-like protein 3

ATGTTGCAGTGCTTAGAAGGGGTTAAGCATTTGTGTGCTTCCCTACTTCGCTGCTGTGATCTTGATTTGTATAGACAATCCAGAGACCTTGAAGATCCTGAAGTTCTTGCAAGGGAGACAGTGTTTAGTGTAAGTGAAATAGAGGCACTTTATGAGCTATTTAAAAAGATTAGCAGCGCAGTAATCGATGACGGGCTGATCAACAAGGAAGAGTTTCAACTGGCATTATTTAAGACAAACAAAAAGGAGAGCTTATTTGCTGATCGGGTATTTGACTTGTTTGACACAAAACATAATGGCATCCTAGGCTTTGAAGAGTTTGCTCGTGCACTCTCTGTATTTCATCCAAATGCCCCTATTGATGATAAGATTGAGTTTTCGTTCCAACTGTACGATCTCAAGCAGCAAGGTTTCATTGAAAGGCAAGAGGTGAAGCAAATGGTAGTGGCGACTCTTGCTGAATCTGGTATGAACCTTTCAGATGATGTTATAGAGAGTATCATCGACAAGACCTTTGAGGAAGCTGATACGAAACATGATGGGAAGATTGACAAGGAAGAGTGGAGAAGTCTTGTACTGCGACATCCATCCCTTTTGAAGAATATGACCCTCCAGTACCTCAAGGACATCACCACTACATTCCCAAGCTTCGTATTTCATTCACGGGTCGAGGATACCTGA

>CSS0035212	PREDICTED: REDICTED:Camellia sinensis galactinol synthase 2-like

ATGTCTCCTGAACTTGTGAGTGCAACCACCAAGGCCACCAGCCTAGTCAAGGCCGCGAGCCTGATGAGCAGAGCTTATGTCACTTTCTTGGCTGGCAATGGTGACTATGTCAAGGGCGTGGTCGGGCTAGCCAAGGGCCTGAGGAAGGTCCACACTGCCTACCCGCTTGTCGTGGCCGTGCTTCCGGATGTTCCGGAGGAACACCGCCATATGCTGGTGGCTCAGGGATGCATAGTCCGTGAGATCGTGCCGGTGTACCCGCCGGAGAACCAGACCCAGTTTGCTATGGCCTACTATGTCATCAACTACTCCAAGCTTCGCATCTGGGAGTTTGTGGAGTACCATAAGATGATATACTTGGACGGAGACATCCAGGTGTTTGAAAACATAGATCACCTCTTTGACAATCCAAATGGCTACTTTTATGCCGTGAAGGACTGTTTTTGTGAAAAAACATGGTCTCACACCCCACAGTACCAGATTGGGTACTGCCAACAGTGCCCTGATAAGGTCCAGTGGCCTGCAGAGTTGGGTCCCCGGCCTCCCCTCTACTTCAATGCTGGCATGTTCGTGTTCGAGCCCAGTCTCTCAATTTATGATGACCTCTTAGAGACCCTCAAAATCACCCCGCCTACCCCTTTTGCTGAGCAGGACTTTTTGAATATGTTCTTTAGGGATGTTTACAAGCCTATCCCACCAATTTACAACCTTGTTTTGGCCATGCTTTGGCGCCACCCAGAGAACATTGAACTCAACAAAGTGAAAGTTGTTCACTATTGTGCCGCGGGATCAAAGCCATGGAGGTATACTGGAAAAGAAGAGAACATGGATAGAAAAGATATCAAGATGCTAGTGAAGAGTTGGTGGGACATATACAATGATGAATCATTGGACTACAAGAGGACTAACATATCACCCATTGAAGGTGAAGCCGACAAAGTCATAGTGACAATGGTGGCAGAGGCCAACGTTGGTCATTACACTCCCGCTCCACCTGCCGCCTAG
