# Supplementary material for: Transcriptome Analysis Reveals Differentially Expressed Genes Involved in Cadmium and Arsenic Accumulation in Tea Plant (Camellia sinensis)
Source: Plants (Basel). 2023 Mar 5;12(5):1182. doi: 10.3390/plants12051182 (PMC10007383; doi:10.3390/plants12051182)
Supplement: Supplementary file 1 [file plants-12-01182-s001.zip › Tables.zip!/Table S5.docx]

| **TFs** | **Total** | **Cd1 *vs.* CK** | | **Total** | **Cd2 *vs.* CK** | | **As1 *vs.* CK** | | **Total** | **As2 *vs.* CK** | |
| --- | --- | --- | --- | --- | --- | --- | --- | --- | --- | --- | --- |
|  |  | **UP** | **Down** |  | **UP** | **Down** | **UP** | **Down** |  | **UP** | **Down** |
| MYB | 23 | 2 | 21 | 14 | 1 | 13 | 0 | 15 | 4 | 0 | 4 |
| WD40 | 1 | 0 | 1 | 1 | 1 | 0 | 0 | 1 | 1 | 1 | 0 |
| bHLH | 16 | 4 | 12 | 7 | 2 | 5 | 4 | 10 | 3 | 0 | 3 |
| AP2 | 33 | 5 | 28 | 27 | 7 | 20 | 5 | 23 | 5 | 1 | 4 |
| Dof | 6 | 5 | 1 | 4 | 2 | 2 | 6 | 0 | 0 | 0 | 0 |
| WRKY | 14 | 0 | 14 | 6 | 1 | 5 | 0 | 1 | 0 | 0 | 0 |
| bZIP | 5 | 0 | 5 | 4 | 1 | 3 | 1 | 2 | 1 | 1 | 0 |
| GRAS | 12 | 2 | 10 | 3 | 2 | 1 | 1 | 3 | 3 | 0 | 3 |
| GRF | 1 | 1 | 0 | 1 | 1 | 0 | 0 | 1 | 0 | 0 | 0 |
| ZF-HD | 1 | 0 | 1 | 1 | 1 | 0 | 0 | 0 | 0 | 0 | 0 |
| ARF | 0 | 0 | 0 | 0 | 0 | 0 | 1 | 1 | 0 | 0 | 0 |
| HSF | 1 | 0 | 1 | 0 | 0 | 0 | 0 | 0 | 0 | 0 | 0 |
| C3H | 0 | 0 | 0 | 0 | 0 | 0 | 1 | 0 | 0 | 0 | 0 |
| TCP | 0 | 0 | 0 | 0 | 0 | 0 | 0 | 0 | 1 | 1 | 0 |
| LSD | 0 | 0 | 0 | 0 | 0 | 0 | 0 | 0 | 0 | 0 | 0 |
| SBP | 1 | 1 | 0 | 0 | 0 | 0 | 0 | 0 | 0 | 0 | 0 |

**Supplementary Table S5 | Number of differentially expressed transcription factors in Cd/As-treated tea roots.**
