# Supplementary material for: Transcriptome Analysis Reveals Differentially Expressed Genes Involved in Cadmium and Arsenic Accumulation in Tea Plant (Camellia sinensis)
Source: Plants (Basel). 2023 Mar 5;12(5):1182. doi: 10.3390/plants12051182 (PMC10007383; doi:10.3390/plants12051182)
Supplement: Supplementary file 1 [file plants-12-01182-s001.zip › Tables.zip!/Table S6.docx]

**Supplementary Table S6 | Expression level of differentially expressed structural genes in Cd/As-treated tea roots.**

| **Gene** | **CK** | **Cd1** | **Cd2** | **As1** | **As2** |
| --- | --- | --- | --- | --- | --- |
| CSS0004240 | 42.91 | 10.65 | 10.84 | 17.61 | 10.12 |
| CSS0030465 | 39.26 | 8.46 | 7.61 | 15.06 | 8.78 |
| CSS0023496 | 60.59 | 20.45 | 21.84 | 17.00 | 8.09 |
| CSS0016059 | 8.15 | 0.88 | 1.07 | 1.36 | 0.42 |
| CSS0020593 | 14.32 | 0.84 | 1.67 | 3.38 | 1.77 |
| CSS0020844 | 46.50 | 18.82 | 20.09 | 15.42 | 13.87 |
| CSS0041012 | 13.12 | 3.17 | 4.77 | 1.93 | 1.20 |
| CSS0047052 | 1.35 | 0.00 | 0.00 | 0.00 | 0.01 |
| CSS0040248 | 34.24 | 5.43 | 11.06 | 11.12 | 11.30 |
| CSS0004486 | 6.58 | 0.05 | 0.00 | 0.00 | 0.06 |
| CSS0028973 | 3.36 | 0.11 | 0.20 | 0.02 | 0.05 |
| CSS0037008 | 1.19 | 0.00 | 0.00 | 0.00 | 0.00 |
| CSS0010197 | 2.95 | 0.08 | 0.00 | 0.00 | 0.00 |
| CSS0020890 | 0.60 | 0.00 | 0.00 | 0.00 | 0.00 |
| CSS0006063 | 6.57 | 1.50 | 0.52 | 0.91 | 0.93 |
| CSS0034513 | 66.82 | 2.79 | 5.00 | 5.31 | 5.47 |
| CSS0028732 | 5.34 | 0.14 | 0.88 | 0.30 | 0.46 |
| CSS0048579 | 9.55 | 0.00 | 0.36 | 0.05 | 0.35 |
| CSS0029000 | 7.97 | 0.38 | 0.38 | 0.47 | 0.76 |
| CSS0049590 | 8.08 | 0.08 | 0.18 | 0.00 | 0.28 |
| CSS0002702 | 8.04 | 0.15 | 0.50 | 0.25 | 0.69 |
| CSS0020354 | 25.40 | 0.71 | 0.63 | 0.23 | 1.47 |
| CSS0039776 | 7.15 | 1.09 | 0.20 | 0.42 | 0.75 |
| CSS0033136 | 112.50 | 20.43 | 15.65 | 13.54 | 33.40 |
| CSS0028763 | 18.09 | 2.51 | 3.01 | 2.45 | 3.91 |
| CSS0043971 | 12.30 | 0.50 | 0.17 | 0.40 | 1.63 |
| CSS0020368 | 2.23 | 0.32 | 0.18 | 0.18 | 0.33 |
| CSS0025316 | 31.96 | 2.27 | 1.52 | 0.40 | 2.76 |
| CSS0045087 | 4.76 | 0.05 | 0.21 | 0.00 | 0.35 |
| CSS0025818 | 33.74 | 4.51 | 6.33 | 6.24 | 8.49 |
| CSS0048993 | 16.07 | 0.27 | 0.23 | 0.17 | 1.55 |
| CSS0009690 | 5.49 | 1.48 | 2.34 | 1.51 | 2.13 |
| CSS0034980 | 7.64 | 0.55 | 1.26 | 0.70 | 1.77 |
| CSS0019841 | 131.14 | 35.21 | 49.98 | 11.05 | 24.97 |
| CSS0035451 | 12.74 | 0.57 | 1.46 | 0.00 | 1.46 |
| CSS0039070 | 3.23 | 0.26 | 0.15 | 0.03 | 0.44 |
| CSS0042374 | 14.45 | 5.15 | 2.54 | 1.04 | 2.87 |
| CSS0004428 | 4.03 | 19.80 | 22.12 | 20.68 | 38.34 |
| CSS0000647 | 13.00 | 1.45 | 47.04 | 1.04 | 50.87 |
| CSS0033791 | 9.99 | 0.82 | 25.72 | 1.06 | 34.74 |
| CSS0050491 | 7.10 | 0.60 | 17.71 | 0.73 | 24.24 |
| CSS0001107 | 39.70 | 6.69 | 91.16 | 5.30 | 104.97 |
| CSS0019367 | 31.25 | 6.63 | 69.94 | 3.13 | 84.12 |
| CSS0006162 | 81.61 | 4.08 | 228.99 | 1.88 | 262.29 |
| CSS0035212 | 82.50 | 10.74 | 215.68 | 6.99 | 247.47 |
