# Supplementary material for: Transcriptome Analysis Reveals Differentially Expressed Genes Involved in Cadmium and Arsenic Accumulation in Tea Plant (Camellia sinensis)
Source: Plants (Basel). 2023 Mar 5;12(5):1182. doi: 10.3390/plants12051182 (PMC10007383; doi:10.3390/plants12051182)
Supplement: Supplementary file 1 [file plants-12-01182-s001.zip › Tables.zip!/Table S7.docx]

| *C. sinensis* | | *A. thaliana* | | E-value | Function description | Source |
| --- | --- | --- | --- | --- | --- | --- |
| Gene locus | Gene name | Gene locus | Gene name |  |  |  |
| CSS0050491 | *CsRS8* | AT5G40390 | *AtRS5* | 0 | Involved in response to drought and cold stress | doi: 10.3390/ijms24010202 |
| CSS0019367 | *CsGolS* | AT2G47180 | *AtGolS1* | 5.6E-149 | Involved in detoxification of heavy metal cadmium | doi:10.1016/j.envexpbot.2023.105217 |
| CSS0033791 | *CsNCED* | AT3G14440 | *AtNCED3* | 4.20E-251 | Involved in detoxification of heavy metal cadmium | doi.org/3389.2020/fpls.00909 |
| CSS0001107 | *CsGolS* | AT2G47180 | *AtGolS1* | 1.10E-147 | Involved in detoxification of heavy metal cadmium | doi:10.1016/j.envexpbot.2023.105217 |
| CSS0035212 | *CsGolS* | AT2G47180 | *AtGolS1* | 5.70E-149 | Involved in detoxification of heavy metal cadmium | doi:10.1016/j.envexpbot.2023.105217 |
| CSS0006162 | *CsHMP* | AT4G08570 | *AtHMP41* | 2.60E-48 | Heavy metal transport/detoxification superfamily protein | - |
| CSS0004428 | *CsCBL7* | AT5G55990 | *AtCBL2/3* | 1.10E-114 | Involved in protecting plants from high Mg2+ | doi: 10.1073/pnas.1420944112 |
| CSS0000647 | *CsERF54* | AT2G20880 | *AtERF53* | 7.90E-53 | Involved in response to drought stress | doi 10.1007/s11103-013-0054-z |

**Supplementary Table 7 |** Functional annotation of eight candidate genes in *C. sinensis.*
