# Supplementary material for: Transcriptome Analysis Reveals Differentially Expressed Genes Involved in Cadmium and Arsenic Accumulation in Tea Plant (Camellia sinensis)
Source: Plants (Basel). 2023 Mar 5;12(5):1182. doi: 10.3390/plants12051182 (PMC10007383; doi:10.3390/plants12051182)
Supplement: Supplementary file 1 [file plants-12-01182-s001.zip › Tables.zip!/Table S9.docx]

**Supplementary Table S9 | Validation of the expression patterns of eight candidate DEGs in Cd/As-treated tea roots.**

|  |  |  | **10d** | | | | **15d** | | | |
| --- | --- | --- | --- | --- | --- | --- | --- | --- | --- | --- |
|  | **CK** | **Standard deviation** | **Cd1** | **Standard deviation** | **As1** | **Standard deviation** | **Cd2** | **Standard deviation** | **As2** | **standard deviation** |
| CSS0050491 | 1.0003 | 0.0234 | 4.0736 | 0.7171 | 4.7701 | 0.7795 | 1.9432 | 0.1658 | 2.9472 | 0.1803 |
| CSS0019367 | 1.0028 | 0.0743 | 0.7247 | 0.0643 | 1.1690 | 0.1297 | 0.3368 | 0.0332 | 1.0385 | 0.1261 |
| CSS0033791 | 1.0017 | 0.0568 | 0.0150 | 0.0012 | 1.5934 | 0.1359 | 0.5504 | 0.1482 | 2.2806 | 0.1391 |
| CSS0001107 | 1.0123 | 0.1603 | 0.0249 | 0.0030 | 1.2363 | 0.0754 | 0.0243 | 0.0022 | 1.1789 | 0.0370 |
| CSS0035212 | 1.0024 | 0.0688 | 0.0598 | 0.0068 | 1.3299 | 0.3091 | 0.0271 | 0.0025 | 3.8145 | 0.5794 |
| CSS0006162 | 1.0045 | 0.0962 | 0.8979 | 0.0933 | 3.2973 | 0.3531 | 0.0578 | 0.0009 | 4.1217 | 0.5536 |
| CSS0004428 | 1.0051 | 0.1006 | 0.6448 | 0.0814 | 1.4323 | 0.2152 | 0.3530 | 0.0464 | 17.8280 | 1.4254 |
| CSS0000647 | 1.0003 | 0.0234 | 4.0736 | 1.4971 | 4.7701 | 1.7795 | 1.9432 | 0.1658 | 2.9472 | 0.1803 |
